# Driving powers of the globalization on the urban ecology, a comparative study

**DOI:** 10.1186/s40068-021-00244-2

**Published:** 2021-12-13

**Authors:** M. Ebrahimi, B. Khalesi, M. R. Mansouri Daneshvar

**Affiliations:** 1 Department of Physical Geography, Hakim Sabzevari University, Sabzevar, Iran; 2 Department of Urban Planning and Design, Mashhad Branch, Islamic Azad University, Mashhad, Iran; 3 Department of Geography and Natural Hazards, Research Institute of Shakhes Pajouh, Isfahan, Iran

**Keywords:** Urbanization, Globalization, Correlation test, Structural analysis, Italy, Japan

## Abstract

**Background:**

The present study investigates the driving effects of globalization on the urban environment in two countries of Italy and Japan, which have the regular amplified economy among the advanced countries. For this purpose, a model with the collaboration of two main subjects of globalization coverage and urbanization and the methodological procedures of correlation test and structural analysis was constructed. A globalization index, namely the Maastricht globalization index (MGI), was assumed based on the integrated values of ten factors [HDI, ITA, GDP, FDI, TEI, GEE, GME, MCS, and IUI] besides three ecological indicators as the baseline of the urban environment, namely carbon dioxide emission (CDE), municipal solid wastes (MSW), and wastewater treatment plants (WTP).

**Results:**

Results revealed the positive associations between globalization and wastewater treatment of urban areas in both countries, exposing the influential role of globalization in connecting the urban population to the sewage plants. The results confirmed the positive role of globalization in decreasing carbon dioxide emissions and overall its practical influences to mitigate urban air pollution. However, the overall globalization effect on urban waste production was estimated differently in both countries.

**Conclusions:**

Based on the MICMAC analysis, only three factors, namely HDI, ITA, GDP, and FDI, can express driving powers and a significant share of globalization coverage. Consequently, enhancing such indicators that belong to globalization’s social and economic domains certainly can act as driver powers to mitigate the environmental issues of urbanization in the study areas.

## Introduction

Nowadays, the world is rapidly developing in terms of globalization and urbanization (Shahbaz et al. 2016). Globalization through the international space and urbanization can change the economic environment (Nedomlelová and Kocourek 2011). It can be explained as the high level of integration and synchronization of national economies, the internationalization of production technology, and the growing flows of services (Marginean 2015). The complex and multidimensional globalization process dominantly focuses on the ratios of international trades, investments, and GDP (Zinkina et al. 2013). As the theoretical implication, the globalization coverage can be indexed using statistical methods, such as the Maastricht Globalization Index (MGI), which has been reported previously by OECD (2008), Martens and Raza (2009), Figge and Martens (2014). The globalization index involves at least some multidisciplinary elements, e.g., social, economic, political, and technological measurements, and it can be used to empirically address manmade impacts (Martens et al. 2010; Zinkina et al. 2013). Nowadays, globalization indices are increasingly used to compare between countries (Figge et al. 2017).

Globalization can influence national changes through each economic sector (e.g., Goldberg and Pavcnik 2007). The present paper investigates the main question of how globalization can influence the urban sector. The research’s motivation relates to the occurrences of the recent global-scale crisis such as climate change and COVID-19 pandemic, which influenced the urban process and life by limiting global trade and flows of people (Yaya et al. 2020). These global changes fundamentally influence human life patterns and activities, especially in urban areas (McMichael 2013). In contrast, the rise in urbanization and the integration of the world economy has facilitated global interconnectedness (Marginean 2015), which could proceed as a mechanism for the transmission of each outbreak (Shrestha et al. 2020). Many researchers, e.g., Jiang and Guan (2017) and Shuai et al. (2017), have reported the relationship between globalization and other aspects of environmental ecosystems such as climate change. It can be seen that the scholars have combined the urbanization processes under the globalization categories (e.g., Khan et al. 2019).

Although academics, politicians, and policymakers have widely debated the impacts of globalization and its various dimensions, there is no consensus regarding globalization’s benefits in each sector, such as urbanization (Sapkota 2010). Investigating the globalization effects on the urban-associated indications is a notable topic that researchers can more interpret because the urban areas should receive a greater and proportional share of the international sustainable development programs in the future (Parnell 2018). It is necessary to provide insightful studies for exploring the interactions between globalization and urban ecology (Fan et al. 2017). A bibliographic assessment of urban globalization researches revealed that the research coverage remains uneven and partial, and a large set of studies has shifted more definitively in local-level findings (Kanai et al. 2018). Hence, the present study investigates the driving effects of globalization on the country-level urban-ecology in two countries, i.e., Italy and Japan, which have different economic and technological potentials. Overall, this paper’s main proposition is that globalization can positively promote the urban ecology, and some essential domains like technological infrastructure can decrease country-level urban pollutions, such as solid wastes.

## Data setting

### Study area

The study areas in this research are confined to two countries of Italy and Japan. Italy, with a total population of 60,298,000 inhabitants, is approximately laid between latitude 36° N to 48° N and longitude 7° E to 18° E. In contrast, Japan, with a total population of 126,265,000 inhabitants, is approximately laid between latitude 30° N to 46° N and longitude 128° E to 147° E. Besides, both countries have different globalized econometrics. For instance, Italy has a mean annual GDP per capita ~33,000 US$ with a human development index of 0.883, while Japan has a mean annual GDP per capita ~40,000 US$ with a human development index of 0.915 (World Bank 2020).

### Preparation of the variables

In the first step, the research variables adopted by the research objective were gained from international databases. In this regard, we assumed two main subjects of globalization and urbanization to obtain a globalization index and urban indications. For this purpose, the globalization index is constructed based on an average standardized value between nine factors (from F1 to F9) in this study (Table 1), which can be classified into four domains of social, economic, political, and technological measurements as expressed as the Maastricht Globalization Index (MGI) by Martens and Raza (2009) and Figge and Martens (2014). On this basis, nine indicators of human development index (HDI), international tourism arrivals (ITA), gross domestic product (GDP), foreign direct investment (FDI), trade of exports and imports (TEI), government education expenditure (GEE), government military expenditure (GME), mobile cellular subscriptions (MCS), and individuals using the Internet (IUI) were considered to produce the MGI.

**Table 1:** Specific indicators for the MGI

Domain	Indicator
Social	[F1] Human development index (HDI) (unitless)
	[F2] International tourism arrivals (ITA) (millions of people)
Economic	[F3] Gross domestic product (GDP) (billions US$)
	[F4] Foreign direct investment (FDI) (% of GDP)
	[F5] Trade of exports and imports (TEI) (% of GDP)
Political	[F6] Government education expenditure (GEE) (% of GDP)
	[F7] Government military expenditure (GME) (% of GDP)
Technological	[F8] Mobile cellular subscriptions (MCS) (per 100 people)
	[F9] Individuals using the Internet (IUI) (% of population)

The majority of the studies have attempted to use the same format of developing globalization indicators such as national GDP, ratios of trade, international tourism, import and export of goods and services (Zinkina et al. 2013), which can be collected directly by official international staffs like the World Bank and other UN organizations. For instance, other globalization indices have been proposed, such as the globalization convergence index (GCI) developed by Martens and Zywietz (2006), The KOF globalization index by Dreher (2006), and the new globalization index (NGI) by Vujakovic (2010) to propose additional insights in the multidimensional concept of globalization (Gygli et al. 2018).

In the current study, variables for globalization subjects were selected based on their relevancy and reasonable linking with five domains of MGI (e.g., Martens et al. 2010), which were selected from 1440 country-level indicators of the World Bank (2020). Three indicators (F10, F11, and F12) are assumed for defining the urban-ecology indications, namely, carbon dioxide emission (CDE), municipal solid wastes (MSW), and wastewater treatment plants (WTP), which can represent urbanization-associated effects in the soil and water environments (Table 2). All the aforementioned data, including globalization and urbanization indicators, were collected from the World development indicators of the World Bank dataset via https://databank.worldbank.org/source/world-development-indicators, which were prepared based on four annual time-series within 2012, 2014, 2016, and 2018, arranged for both countries of Italy and Japan.

**Table 2:** Specific indicators for urban-ecology indicators

Domain	Indicator
Ecological	[F10] Carbon dioxide emission (CDE) (megatons)
	[F11] Municipal solid wastes (MSW) (thousand tons per year)
	[F12] Wastewater treatment plants (WTP) (% of urban population)

Furthermore, three variables of HDI, MSW, and WTP were supported from other global datasets. As an integrated and standardized indicator of social status and the people’s capabilities, the annual values of the HDI were assumed through the development of a country and were accessed from the United Nations Development Programme (UNDP) via http://hdr.undp.org/en/content/human-development-index-hdi. Total generation of the municipal solid wastes was gained from the generated waste statistics of the Organization for Economic Cooperation and Development (OECD) database via https://stats.oecd.org/Index.aspx. Besides, wastewater treatment plants data was gained from the OECD statistics of sewage treatment connection rates via https://data.oecd.org/water/waste-water-treatment.htm.

## Methodology

### Research model

**Fig. 1:**
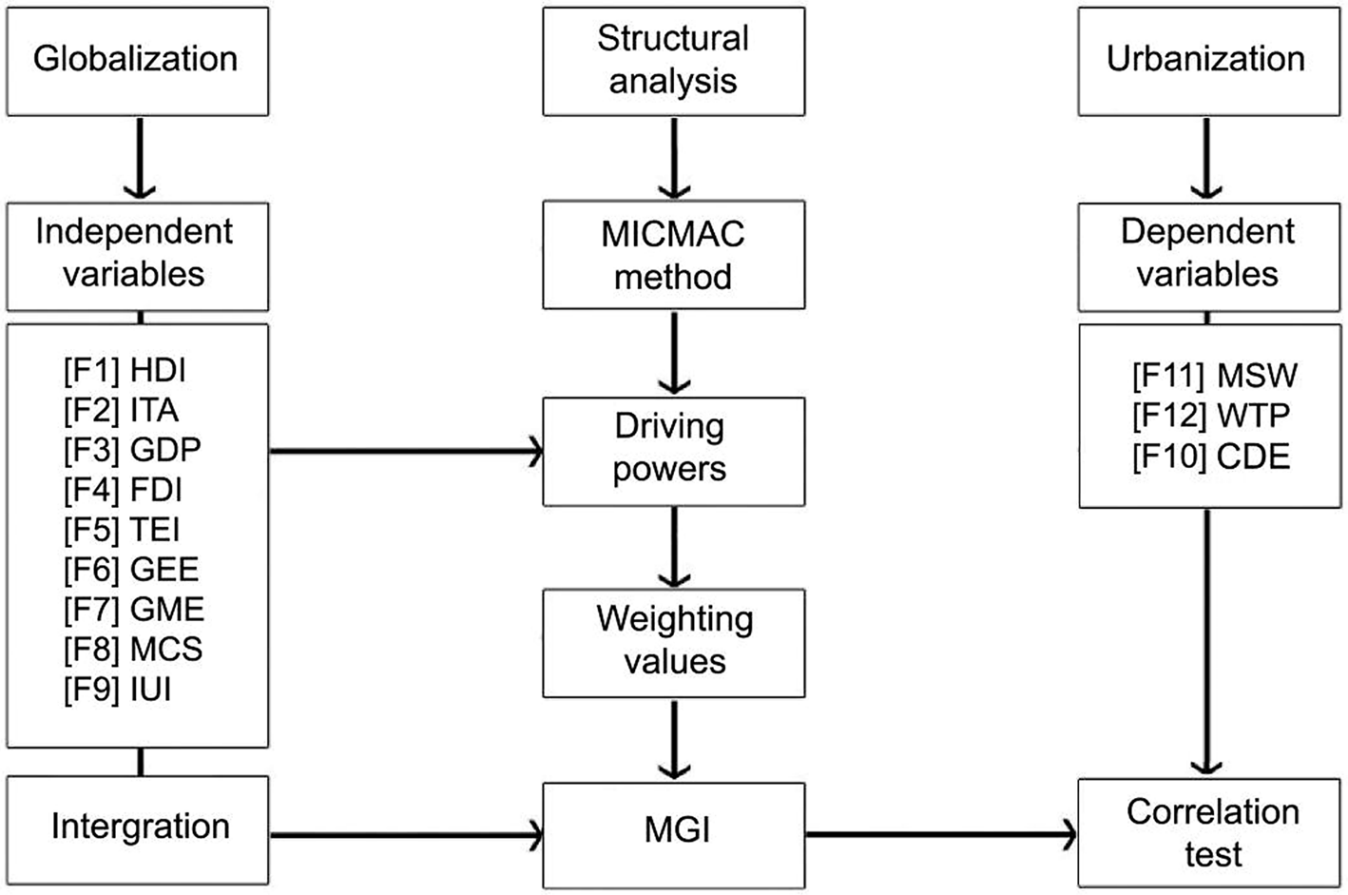
Research model

For this purpose, we constructed a model collaborating with two main subjects of globalization and urbanization and the methodological procedures of correlation test and structural analysis (Fig. 1). Based on the model, the obtained variables can be classified as two distinct groups of independent variables (from F1 to F9 as mentioned above for globalization subject) and dependent variables (F10, F11, and F12 as mentioned above for urbanization subject). In the first step of the model, a structural analysis, namely MICMAC, is considered to identify the key driving powers between globalization factors. The driver powers are the factors that explain a great share of globalization coverage and can be categorized as the powerful and effective variables influencing urbanization in the study areas within the time periods. We should consider the driving powers as weighting values for each factor to obtain an accurate and comparable MGI value between the countries. In the second step, the ten factors of globalization are integrated based on the defined equations (1 and 2) to convert all factor values into a standardized indicator, namely MGI. To examine the significance of our estimations, we need to discover the globalization effects on the urban-ecology. For this justification and in the third step, the relations of MGI and its related factors with urbanization are investigated using the correlation test. The correlation test output will show the significant negative and positive relationships between the aforementioned independent and dependent variables.

### Structural analysis

After the data preparation, the main procedure to define dependence or driving powers between MGI-related factors is assumed as an Interpretive Structural Modeling (ISM), performed by MICMAC analysis, which refers to cross-multiplication impacts (Lim et al. 2017; Raut and Gardas 2018). This procedure follows four categories based on the dependence and driving power values of the variables in a Final Reachability Matrix (FRM) (Wang et al. 2018; Fathi et al. 2019). This procedure is carried out in some steps as below (Faisal and Talib 2016; Iyengar et al. 2017; Ghobakhloo 2020):

1. Establishing contextual relationships among each pair of variables followed by verbal values of V (i determines j), A (i is determined by j), X (i and j determine each other), and O (i and j are unrelated) to create a Structural Self-Interaction Matrix (SSIM);
2. Establishing the Initial Reachability Matrix (IRM), as a binary matrix, by replacing verbal values with digital values 0 (for A and O) and 1 (for V and X);
3. Establishing the Final Reachability Matrix (FRM), developed by subjecting the interrelationships within the IRM to estimate driving powers and dependence powers (Dev and Shankar 2016, Thirupathi and Vinodh 2016, Fathi et al. 2019, Ghobakhloo 2020), which is represented in a quartile plot. Eventually, the structural analysis reveals the driving powers between the globalization index variables, leading to effective factors for defining the weighting values by estimating the MGI values.

### Equations

After obtaining the relevant variables for MGI, each factor value (*Vi*) is transformed to standardized value (*Xi*) from zero to hundred using the below Eq. 1 (Figge et al. 2017):

1 $$X_{i} = \frac{{(V_{i} - V_{\min } )}}{{(V_{\max } - V_{\min } )}} \times 100$$

where, *Vi* is the factor value in each year, and *Vmin* and *Vmax* are the minimum and maximum values of each factor for both countries within multiple years.

Finally, the MGI value is estimated by multiplying all standardized values (*Xi*) with weighting values (*Wi*) to obtain the globalization index using the below Eq. 2:

2 $$MGI = \frac{{\sum {(W_{i} \times X_{i} )} }}{{\sum {(W_{i} )} }}$$

where, *Wi* is the weighting value for each factor, which is estimated based on the driving powers and the research topic of globalization effects on the urban ecology in the final reachability matrix (FRM). The systematic method to obtain the assigned weights, entitled as driving powers, has been interpreted in sub-section 3.2. structural analysis. Deriving powers can prioritize the factors regarding the research aim and topic. Ultimately, the MGI values closer to a hundred can denote more globalization or internationalization status among the compared countries within the given time windows.

The proposed procedures, i.e., the equations for standardizing factor values and calculating globalization index, can be generalized for other new and more countries. However, the weighting values in equation 2 should be re-evaluated based on the research topics and aims. Meanwhile, the data for the urbanization indicators are obtained in the quality-controlled format directly from the global database and then are normalized using SPSS software.

### Correlation analysis

Scholars perform the globalization indices conventionally correlated by each given subject based on the statistical analysis (e.g., Martens et al. 2010, Lim and Tsutsui 2011, Figge et al. 2017). In the last step, a correlation test is considered between the mean values of MGI, as a rate of the globalization effect, and four independent variables, as the urbanization indicators, in each country within four periods (2012, 2014, 2016, and 2018).

## Results and discussion

### Analysis of data

This section estimates the raw and pmyrocessed variables to detect the effective role of globalization indicators in the urban environment, i.e., municipal solid waste production and wastewater treatment plants. For this purpose, the values of all variables, i.e., ten globalization factors besides two urbanization factors, were gained in Tables 3 and 4, revealing for Italy and Japan within time intervals 2012, 2014, 2016, and 2018. The urban-associated waste production revealed a decrease from 45,234 to 42,894 thousand tons in Japan during 2012–2018, while it exposed an increase from 29,994 to 30,165 thousand tons in Italy in the exact times. The urban population connected to wastewater treatment plants increased from 76.30 to 78.80 % in Japan and from 60.83 to 63.00 % in Italy.

**Table 3:** The values of 12 indicators in Italy during 2012–2018

Indicator	2012	2014	2016	2018
[F1] Human development index (HDI)	0.874	0.874	0.878	0.883
[F2] International tourism arrivals (ITA)	46.36	48.58	52.37	61.57
[F3] Gross domestic product (GDP)	2087	2159	1876	2092
[F4] Foreign direct investment (FDI)	0.33	0.95	0.75	1.90
[F5] Trade of exports and imports (TEI)	55.65	55.32	55.37	60.35
[F6] Government education expenditure (GEE)	4.06	4.06	3.82	4.04
[F7] Government military expenditure (GME)	1.44	1.29	1.34	1.34
[F8] Mobile cellular subscriptions (MCS)	162.31	148.84	141.69	137.47
[F9] Individuals using the Internet (IUI)	55.83	55.64	61.32	74.39
[F10] Carbon dioxide emission (CDE)	401	350	356	348
[F11] Municipal solid wastes (MSW)	29,994	29,652	30,112	30,165
[F12] Wastewater treatment plants (WTP)	60.83	61.00	62.50	63.00

**Table 4:** The values of 12 indicators in Japan during 2012–2018

Indicator	2012	2014	2016	2018
[F1] Human development index (HDI)	0.895	0.904	0.910	0.915
[F2] International tourism arrivals (ITA)	8.36	13.41	24.04	31.19
[F3] Gross domestic product (GDP)	6203	4850	4923	4955
[F4] Foreign direct investment (FDI)	1.90	2.84	3.63	3.20
[F5] Trade of exports and imports (TEI)	30.64	37.55	31.54	36.82
[F6] Government education expenditure (GEE)	3.69	3.59	3.19	3.18
[F7] Government military expenditure (GME)	0.97	0.97	0.94	0.94
[F8] Mobile cellular subscriptions (MCS)	109.89	123.16	130.60	139.20
[F9] Individuals using the Internet (IUI)	79.50	89.11	93.18	84.59
[F10] Carbon dioxide emission (CDE)	1305	1263	1203	1136
[F11] Municipal solid wastes (MSW)	45,234	44,317	43,170	42,894
[F12] Wastewater treatment plants (WTP)	76.30	77.60	78.30	78.80

The various urbanization trends in the two countries need to be investigated by detecting effective and driver powers. For example, and as a primitive investigation, the values for some globalization-associated factors such as gross domestic product (GDP) and mobile cellular subscriptions (MCS) have a different trend in both countries during 2012–2018. For example, the annual amounts of GDP in 2018 have been recorded as equal to 4955 (2092) billion US dollars for Japan (Italy), while the annual amounts of TEI in 2018 have been observed equally to 36.82 (60.35) percentage of GDP for Japan (Italy). Some other factors have similar increasing or decreasing trends within four-time intervals. For example, HDI values increased from 0.871 (0.885) to 0.883 (0.915) in Italy (Japan), revealing the improved human development and welfare conditions in both countries. Moreover, CDE values decreased from 401 (1305) to 348 (1136) megatons in Italy (Japan), revealing the improved air quality by mitigating carbon dioxide emissions in both countries.

### Estimation of the driving powers

Before estimating MGI, we should define the driving powers among the nine factors, categorized as the weighting values. For this purpose, the MICMAC analysis is assumed through interpretive structural modeling. Hence, ten MGI factors were considered to estimate the contextual relationships based on the multiple expert judgments. The outputs were gained in the SSIM as shown in Table 5, which were considered to produce further converted values into initial and final reachability matrixes (IRM and FRM) as depicted in Tables 6 and 7.

**Table 5:** Structural self-interaction matrix (SSIM) between the MGI factors (F = 9)

Factors	[F1]	[F2]	[F3]	[F4]	[F5]	[F6]	[F7]	[F8]	[F9]
[F1]	–	X	A	X	O	V	O	V	X
[F2]	X	–	V	X	V	X	O	O	X
[F3]	V	A	–	A	A	V	V	V	V
[F4]	X	X	V	–	V	O	X	V	V
[F5]	O	A	V	A	–	A	X	X	V
[F6]	A	X	A	O	V	–	O	X	A
[F7]	O	O	A	X	X	O	–	O	O
[F8]	A	O	A	A	X	X	O	–	X
[F9]	X	X	A	A	A	V	O	X	–

**Table 6:** Initial reachability matrix (IRM) between the MGI factors (F = 9)

Factors	[F1]	[F2]	[F3]	[F4]	[F5]	[F6]	[F7]	[F8]	[F9]
[F1]	–	1	0	1	0	1	0	1	1
[F2]	1	–	1	1	1	1	0	0	1
[F3]	1	0	–	0	0	1	1	1	1
[F4]	1	1	1	–	1	0	1	1	1
[F5]	0	0	1	0	–	0	1	1	1
[F6]	0	1	0	0	1	–	0	1	0
[F7]	0	0	0	1	1	0	–	0	0
[F8]	0	0	0	0	1	1	0	–	1
[F9]	1	1	0	0	0	1	0	1	–

**Table 7:** Final reachability matrix (FRM) between the MGI factors (F = 9)

Factors	[F1]	[F2]	[F3]	[F4]	[F5]	[F6]	[F7]	[F8]	[F9]	Driving power
[F1]	–	1	0	1	0	1	0	1	1	5
[F2]	1	–	1	1	1	1	0	0	1	6
[F3]	1	0	–	0	0	1	1	1	1	5
[F4]	1	1	1	–	1	0	1	1	1	7
[F5]	0	0	1	0	–	0	1	1	1	4
[F6]	0	1	0	0	1	–	0	1	0	3
[F7]	0	0	0	1	1	0	–	0	0	2
[F8]	0	0	0	0	1	1	0	–	1	3
[F9]	1	1	0	0	0	1	0	1	–	4
Dependence power	4	4	3	3	5	5	3	6	6	39

**Fig. 2:**
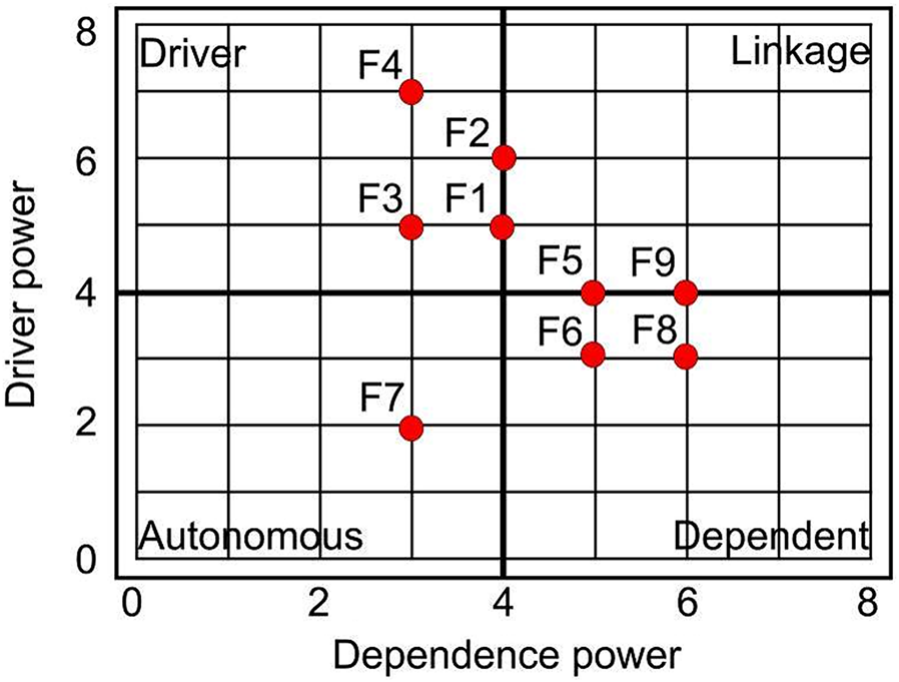
Driving and dependence power of each MGI factor as an output of the MICMAC analysis

In this regard, the SSIM matrix was converted into binary matrixes, which eventually can assist in the cluster of the variables according to the driving and dependence powers. Based on the FRM matrix, all MGI factors were transited into the driving power (total number of factors in row cells in FRM) and dependence power (total number of factors in column cells in FRM). Four different clusters of factors, namely autonomous (without dependence), dependent, linkage (mid driver), and driving powers, can be plotted in Fig. 2, following the MICMAC analysis. On this basis, four factors, namely [F1] human development index (HDI), [F2] international tourism arrivals (ITA), [F3] gross domestic product (GDP), and [F4] foreign direct investment (FDI), can be clustered as driving powers of globalization, which can directly influence each specific subject. This fact is in accordant with previous works. For example, as mentioned by (Zinkina et al. 2013), the multidimensional globalization process dominantly focuses on the ratios of international trades, investments, and GDP. The driving power values are assumed as weighting values for each factor through integrating and estimating the MGI.

### Estimation of the MGI

According to Eqs. 1 and 2, the MGI values were produced in Table 8, revealing the overall decreased globalization coverage for Italy from 2012 to 2018 with values 40.77 and 58.54. Similarly, regular increased globalization coverage was observed for Japan from 2010 to 2018 with 39.21 and 58.49. On this basis, the globalization stories in both countries are similar, depending on the regular amplifying MGI values and components such as the trade levels. The similarity of globalization, particularly in 2018, as estimated 58.54 and 58.49 for Italy and Japan, can be related to the enhanced technological domain, especially through mobile cellular subscriptions and individuals using the Internet. In the next section, we should correlate the urban-ecology indicators with globalization-associated factors to expose the positive or negative impact of globalization on the ecological domain of the study areas.

**Table 8:** The estimated MGI values in Italy and Japan during 2012–2018

Year	MGI	
	Italy	Japan
2012	40.77	39.21
2014	41.38	50.69
2016	40.71	56.48
2018	58.54	58.49

### Correlation tests

The Pearson correlation tests between this study’s dependent and independent variables were estimated based on four times (N=4). The primitive but actual correlation result revealed the positive associations (R=0.71 and 0.99) between globalization (MGI) and wastewater treatment of urban areas in both countries of Italy and Japan, exposing the effective role of globalization on the connecting of urban population to the sewage plants during time-intervals (2010–2018) (Table 9). Contrarily, the result revealed the negative associations (R=− 0.44 and − 0.91) between globalization (MGI) and carbon dioxide emissions in both countries, exposing the sustainable role of globalization in controlling greenhouse gas emissions and urban air pollution. The moderated effect of globalization on the ecological domain of air pollution has been investigated previously in several works but without bolding. For instance, Shahbaz et al. (2018, 2017) and Meng et al. (2016) have investigated the role of the overall globalization index and its sub-domains in the air quality indices, but our results confirmed that if globalization is high, then the carbon dioxide emissions and air pollution become low. The correlation results in Table 9 depends on the exact values of dependent and independent variables for the given study areas of the present study and cannot be generalized for other countries.

**Table 9:** The correlation results between dependent and independent variables in Italy and Japan within four time intervals (N = 4)

Indicators	Test	Italy			Japan		
		[F10] CDE	[F11] MSW	[F12] WTP	[F10] CDE	[F11] MSW	[F12] WTP
MGI	R	− 0.44	0.50	0.71	− 0.91	− 0.98	0.99
	Sig	0.56	0.50	0.29	0.09	0.02	0.01
[F1] HDI	R	− 0.54	0.74	0.95	− 0.97	− 0.99	1.00
	Sig	0.46	0.26	0.05	0.03	0.01	0.00
[F2] ITA	R	− 0.63	0.62	0.92	− 0.99	− 0.97	0.95
	Sig	0.37	0.38	0.08	0.01	0.03	0.05
[F3] GDP	R	0.06	− 0.60	− 0.49	0.66	0.78	− 0.86
	Sig	0.94	0.40	0.51	0.34	0.22	0.14
[F4] FDI	R	− 0.73	0.31	0.74	− 0.77	− 0.92	0.91
	Sig	0.27	0.69	0.26	0.23	0.08	0.09
[F5] TEI	R	− 0.36	0.55	0.70	− 0.42	− 0.35	0.52
	Sig	0.64	0.45	0.30	0.58	0.65	0.48
[F6] GEE	R	0.22	− 0.43	− 0.46	0.93	0.98	− 0.92
	Sig	0.78	0.57	0.54	0.07	0.02	0.08
[F7] GME	R	0.92	0.43	− 0.26	0.95	0.94	− 0.88
	Sig	0.08	0.57	0.74	0.05	0.06	0.12
[F8] MCS	R	0.90	− 0.38	− 0.89	− 0.97	− 0.98	0.99
	Sig	0.10	0.62	0.11	0.03	0.02	0.01
[F9] IUI	R	− 0.50	0.69	0.89	− 0.34	− 0.59	0.60
	Sig	0.50	0.31	0.11	0.66	0.41	0.40

However, the overall globalization effect on urban waste production was estimated differently in both countries. The correlation result revealed the negative relation (R = − 0.98) between globalization (MGI) and the production of municipal wastes in Japan, while this relation was estimated as positive (R = 0.50) in Italy. This finding revealed the different effects of the globalization index on urban waste production, possibly due to the different ecological domains of the mentioned countries. Hence, for investigating the complicated relationships, the correlations were tested between urban indications and ten MGI components (i.e., factors from F1 to F9).

Results revealed the negative correlations (R from − 0.35 to − 0.99) between MSW and six globalization factors, including HDI, ITA, FDI, TEI, MCS, and IUI in Japan, while the negative relations (R from − 0.38 to − 0.60) were observed in Italy for entirely different factors, namely GDP, GEE, MCS, and CDE. This fact reveals that the globalization contribution to decreasing municipal wastes in Japan is significantly affected by social and economic infrastructures, while in Italy, the mitigating role of globalization components has not been sufficient meaningfully. On the other hand, positive correlations (R from 0.52 to 1.00) were observed between WTP and six abovementioned globalization factors, i.e., HDI, ITA, FDI, TEI, MCS, and IUI in Japan. Also, similar positive relations with weaker results (R from 0.30 to 0.95) were observed between the WTP and the abovementioned globalization components, excluding MCS, in Italy. Hence, the social and economic domains of globalization can enhance the urban environment in both countries. These results exactly revealed the declining role of globalization and its technological components, such as the usage of mobile and internet networks, on the production of urban solid wastes in Japan. Niebel (2018) confirmed the effect of the technological level of globalization, measured by mobile and internet usage, on enhancing economic growth, and Asongu et al. (2018) suggested this component as a mitigating measure of carbon dioxide emissions at the country level.

On the other hand, it can be anticipated that by enhancing globalization’s social and economic domains, the connecting urban population to wastewater treatment plants would be increased at the country level. Contrarily, globalization’s political domain, such as the GEE and GME, may be effective in declining wastewater treatment in both countries. Different roles of globalization’s domains have been reported in other recent researches. For instance, Suki et al. (2020) have investigated the different impacts of globalization’s economic, social, and political role in the change of air pollution.

It should be noted that the assigned weights of driving factors are only weighting values obtained from the final reachability matrix (FRM), which are used to estimate the MGI value (based on Eq. 2) and have no relationship with the correlation results in table 9. The correlation results have been estimated to examine the relationships between raw values of independent variables (F1–F9 in addition to calculated MGI) and dependent variables (F10, F11, and F12).

## Discussion

Based on our results, four factors, namely human development index (HDI), international tourism arrivals (ITA), gross domestic product (GDP), and foreign direct investment (FDI), can express strong driving powers of globalization and can explain a significant share of its coverage. Consequently, enhancing the indicators that belong to globalization’s social and economic domains certainly can act as driver powers to mitigate the environmental issues of urbanization in the study areas. According to literature, globalization remains in the first place a very strong and powerful economic phenomenon, where the countries with higher levels of income per capita show also higher levels of human development (Bednářová et al. 2011), i.e., the level of economic development, e.g., GDP and FDI, is reflected in the higher levels of HDI. In recent research, short and long-term coefficients confirm that the social and economic dimensions of globalization are responsible for mitigating environmental issues (Mehmood 2021).

Some researchers have exposed strong relationships between urban-associated indications and environmental changes, e.g., carbon dioxide emissions and electric power consumption, which the urban population can influence. For instance, in the urbanization process, population growth is a notable issue, directly or indirectly affecting land degradation, increases in energy consumption, high Greenhouse Gas (GHG) emission, and global pollution (Ghanbari and Daneshvar 2020). Urbanization in low-income countries could not reveal a significant effect on environmental change. In vice versa, it can mitigate air pollution in high-income countries (Fang et al. 2012). Hence, urban population shares in the study areas, where the share of urban population from the total population are estimated as 70 and 92% for Italy and Japan, respectively (World Bank 2020), can explain our results. It means that globalization can reveal a moderator role in the urban environment (like decreasing waste or increasing wastewater treatment in Japan) when the urban population contributes to each country’s total population. Contrarily, the lower share of Italy’s urban population has not reflected globalization’s strong role in promoting the urban environment.

Consequently, driving powers of globalization, e.g., human development index, international tourism arrivals, gross domestic product, and foreign direct investment, can enhance the urban ecology in such advanced economics, which have an overwhelmed urbanization status. As the economic level advances, its national strategies need more measures to mitigate urban and environmental pollutions (Mehmood 2021). Among such countries, economic factors like GDP and FDI can increase environmental pollution, but at a high level of economics, those can subsidize environmental pollution due to green technology and environment-friendly regulations (Ulucak et al. 2020, Ahmed and Le 2020).

Although recent views about the pandemic situation have represented the declining consequences on the global human health and economy (Ibn-Mohammed et al. 2021), our results have only bolded the social and technological values of globalization, which can positively promote the urban ecology, such as declining solid wastes in country-level and during a time interval of 2012–2018. Some of the globalization dimensions as drivers of sustainable development goals were non-realistic in the pandemic situation (Naidoo and Fisher 2020). However, there is no full answer to the relationship between pandemic effects and following deepening globalization or de-globalization (Sułkowski 2020). Moreover, other recent papers revealed that the future world economy would need even more globalization (Contractor 2021) due to scientific collaboration on sustainable goals (Chapman and Tsuji 2020) and integration of advancements in technology (McKenzie 2020). Hence, further research can examine the interactional impacts of globalization dimensions and urban ecology under the shed of pandemic situations, particularly in 2018–2022.

## Conclusion

In this paper, the globalization effects on ecology were investigated. This study’s motivation relates to the recent global-scale crisis occurrences such as climate change. For this purpose, a model with the collaboration of two main subjects of the globalization coverage and urban environment and the methodological procedures of correlation test and structural analysis was constructed. A globalization index, MGI, was assumed based on integrated values between ten factors besides two urban indicators as the urban environment’s baseline regarding municipal solid wastes and wastewater treatment plants.

The constant correlation revealed the positive associations between MGI and wastewater treatment of urban areas in Italy and Japan, exposing the influential role of globalization components in connecting the urban population to the sewage plants. Moreover, the results revealed the declining role of globalization and its technological components, such as the usage of mobile and internet networks, in the production of urban solid wastes in Japan. On the other hand, it can be anticipated that by enhancing globalization’s social and economic domains, the connecting urban population to wastewater treatment plants would be increased at the country level. Eventually, the paper confirmed the positive role of globalization in decreasing carbon dioxide emissions and its practical influences to mitigate urban air pollution. The main implication of these results is defining the substantial role of social and economic domains of globalization in mitigating urban environmental issues, which can be interpreted sufficiently in future works by a large sample of case studies and time series.

## Data Availability

The data that support the findings of this study are available from the corresponding author upon request.

## Abbreviations

CDE: Carbon dioxide emission
FDI: Foreign direct investment
FRM: Final reachability matrix
GDP: Gross domestic product
GEE: Government education expenditure
GME: Government military expenditure
GHG: Greenhouse gas
HDI: Human development index
IRM: Initial reachability matrix
ISM: Interpretive structural modeling
ITA: International tourism arrivals
IUI: Individuals using the internet
MCS: Mobile cellular subscriptions
MGI: Maastricht globalization index
MICMAC: Matrice d’Impacts Croisés Multiplication Appliquée á un Classement
MSW: Municipal solid wastes
OECD: Organization for Economic Cooperation and Development
SSIM: Structural Self-Interaction Matrix
TEI: Trade of exports and imports
UNDP: United Nations Development Programme
WTP: Wastewater treatment plants
